# Increasing transmission of dengue virus across ecologically diverse regions of Ecuador and associated risk factors

**DOI:** 10.1371/journal.pntd.0011408

**Published:** 2024-01-31

**Authors:** Leah C. Katzelnick, Emmanuelle Quentin, Savannah Colston, Thien-An Ha, Paulina Andrade, Joseph N. S. Eisenberg, Patricio Ponce, Josefina Coloma, Varsovia Cevallos

**Affiliations:** 1 Viral Epidemiology and Immunity Unit, Laboratory of Infectious Diseases, National Institute of Allergy and Infectious Diseases, National Institutes of Health, Bethesda, Maryland, United States of America; 2 Centro de Investigación en Salud Pública y Epidemiología Clínica (CISPEC), Facultad de Ciencias de la Salud Eugenio Espejo, Universidad UTE, Quito, Ecuador; 3 Department of Epidemiology, School of Public Health, University of Michigan, Ann Arbor, Michigan, United States of America; 4 Division of Infectious Diseases and Vaccinology, School of Public Health, University of California, Berkeley, Berkeley, California, United States of America; 5 Centro de Investigación en Enfermedades Infeciosas y Vectoriales (CIREV), Instituto Nacional de Investigación en Salud Pública (INSPI), Quito, Ecuador; The Pennsylvania State University, UNITED STATES

## Abstract

The distribution and intensity of viral diseases transmitted by *Aedes aegypti* mosquitoes, including dengue, have rapidly increased over the last century. Here, we study dengue virus (DENV) transmission across the ecologically and demographically distinct regions or Ecuador. We analyzed province-level age-stratified dengue incidence data from 2000–2019 using catalytic models to estimate the force of infection of DENV over eight decades. We found that provinces established endemic DENV transmission at different time periods. Coastal provinces with the largest and most connected cities had the earliest and highest increase in DENV transmission, starting around 1980 and continuing to the present. In contrast, remote and rural areas with reduced access, like the northern coast and the Amazon regions, experienced a rise in DENV transmission and endemicity only in the last 10 to 20 years. The newly introduced chikungunya and Zika viruses have age-specific distributions of hospital-seeking cases consistent with recent emergence across all provinces. To evaluate factors associated with geographic differences in DENV transmission potential, we modeled DENV vector risk using 11,693 *Aedes aegypti* presence points to the resolution of 1 hectare. In total, 56% of the population of Ecuador, including in provinces identified as having increasing DENV transmission in our models, live in areas with high risk of *Aedes aegypti*, with population size, trash collection, elevation, and access to water as important determinants. Our investigation serves as a case study of the changes driving the expansion of DENV and other arboviruses globally and suggest that control efforts should be expanded to semi-urban and rural areas and to historically isolated regions to counteract increasing dengue outbreaks.

## Author summary

The factors driving the increasing burden of arboviruses like dengue virus are not fully understood. In this study, we measured changes in dengue virus transmission intensity and arbovirus disease risk across Ecuador, an ecologically and demographically diverse South American country. We found that differences in the distribution of dengue cases could be explained by changes in transmission of dengue virus over time: transmission was limited to coastal provinces with large cities between 1980–2000, expanding thereafter to higher elevation areas and ecologically suitable but previously geographically and socially isolated provinces. We also used species distribution mapping to show that both urban and rural areas in Ecuador are at medium to high risk for *Aedes aegypti* presence, with population size, trash removal, elevation, and access to water as strong predictors. Our investigation reveals changes driving the expansion of dengue virus and other arboviruses globally and provides an approach for identifying areas at early stages of establishing endemic transmission that should be targeted for intense preventative efforts to avert future epidemics.

## Introduction

Arboviruses transmitted by the *Aedes aegypti* mosquito (Culicidae), including the flaviviruses yellow fever virus, dengue viruses 1–4 (DENV1-4), and Zika virus (ZIKV) and alphavirus chikungunya virus (CHIKV), infect millions of people globally each year and cause a spectrum of life-threatening diseases with long-term sequelae, including hemorrhagic fevers, arthritis, and severe congenital abnormalities [1,2]. In the 19^th^ and 20^th^ centuries, explosive epidemics of arboviral disease primarily affected large population centers in tropical regions but in the 20^th^ into the 21^st^ century, arboviruses transmission worsened in previously affected areas and expanded into new regions [3–5]. As a result, dengue is one of the few tropical diseases with an increasing global burden over the last 20 years [6]. The distribution of other arboviruses has also increased over the last ten years as ZIKV and CHIKV were introduced to the Americas and caused back-to-back continent-wide pandemics [7,8].

Tracking factors driving the global expansion of arboviruses like DENV is limited by the quality of disease surveillance. In many regions, dengue incidence remains low but whether this is attributable to low reporting rates as opposed to low disease burden is difficult to determine. Recent and historical work suggests the force of infection (FoI), defined as the per-capita rate at which susceptible individuals become infected in a population within a given period, can be directly estimated from age-stratified incidence data and provides a useful measure of transmission intensity [9–14]. The age distribution of incidence is a more accurate measure of transmission intensity than aggregate case count data, and in general reflects transmission intensity estimates made using gold-standard seroprevalence data [9,12,15]. When longitudinal age-stratified seroprevalence or incidence data are available, it is possible to estimate changes in the FoI over time, as originally demonstrated for Hepatitis A [16] and numerous times for DENV [11,13,17–21].

The drivers of arboviral disease can be studied within Ecuador, an ecological and demographically diverse South American country with multiple distinct regions with reported arbovirus transmission [22]. Previous studies in urban centers in Ecuador have shown that household water access and storage methods, crowding, housing condition, garbage collection, and knowledge of dengue are strong local predictors of *Aedes aegypti* pupae presence and density as well as dengue cases [23–27]. Even within these urban areas, hyper-local hotspots have been identified, indicating not all neighborhoods are similarly affected each year [24,28,29]. Dengue cases and vector densities in both urban and rural communities along the Eastern coastal region of Ecuador are affected by seasonal and inter-annual climate effects, with strong predictors including precipitation and minimum temperature [23–25]. Fewer studies have been conducted in the more remote Amazon provinces of Ecuador, although vector mapping studies have suggested suitable conditions for *Aedes aegypti* in parts of this region [30].

Ecuador has a long history of arbovirus transmission, with the first case of yellow fever recorded in 1740 and the first outbreak in 1842 [31,32]. Between 1946 and 1970, eradication campaigns targeted the vectors for yellow fever and malaria, resulting in a major reduction in *Aedes* populations. *Aedes aegypti* soon returned, followed by the re-introduction of the four DENV serotypes [3]. National dengue outbreaks occurred in Ecuador in 1996, 2000, 2005, 2010, 2012, and 2014 and since 2000, the four DENV serotypes have been detected in all regions [33,34]. Phylogenetic analyses have shown both recent and historical introductions of DENV1, DENV2, and DENV4 in Ecuador that likely originated from Venezuela and Colombia, while chikungunya was likely introduced through the Caribbean [35–37].

Here, we modeled age-stratified dengue incidence data from 2000–2019 to estimate changes in the DENV transmission intensity for nearly 80 years and across provinces in Ecuador. We complement this analysis by evaluated factors associated with geographic differences in vector suitability in the last ten years by modeling 11,693 *Aedes aegypti* presence points. We identify provinces at different stages of establishing endemic DENV transmission as well as areas with high vector risk.

## Materials and methods

### Ethics statement

Human subjects data sets were from anonymized public health surveillance data collected by the Ecuadorian Ministry of Health as part of routine care. The Instituto Nacional de Estadística y Censos (INEC) data used in this study is freely available from an institutional website and is completely anonymous [38]. According to the international good clinical and research practice and in accordance with Ecuadorian legislation on clinical investigation, ethical approval by an institutional review board was not required. The public surveillance data collected by the Ecuador Ministry of Health (Sistema Nacional de Vigilancia en Salud Pública Ecuador, ViEpi data [39,40]) was made available for research purposes without confidential information (anonymous), under the "Organic Law of Transparency and Access to Public Information" (Article 4 of Ley Orgánica de Transparencia y Acceso a la Información Pública, LOTAIP, Law 24 of Article 81 of the Ecuadorian Constitution).

### Geography, climate, and demographic characteristics of Ecuador

**Fig 1:**
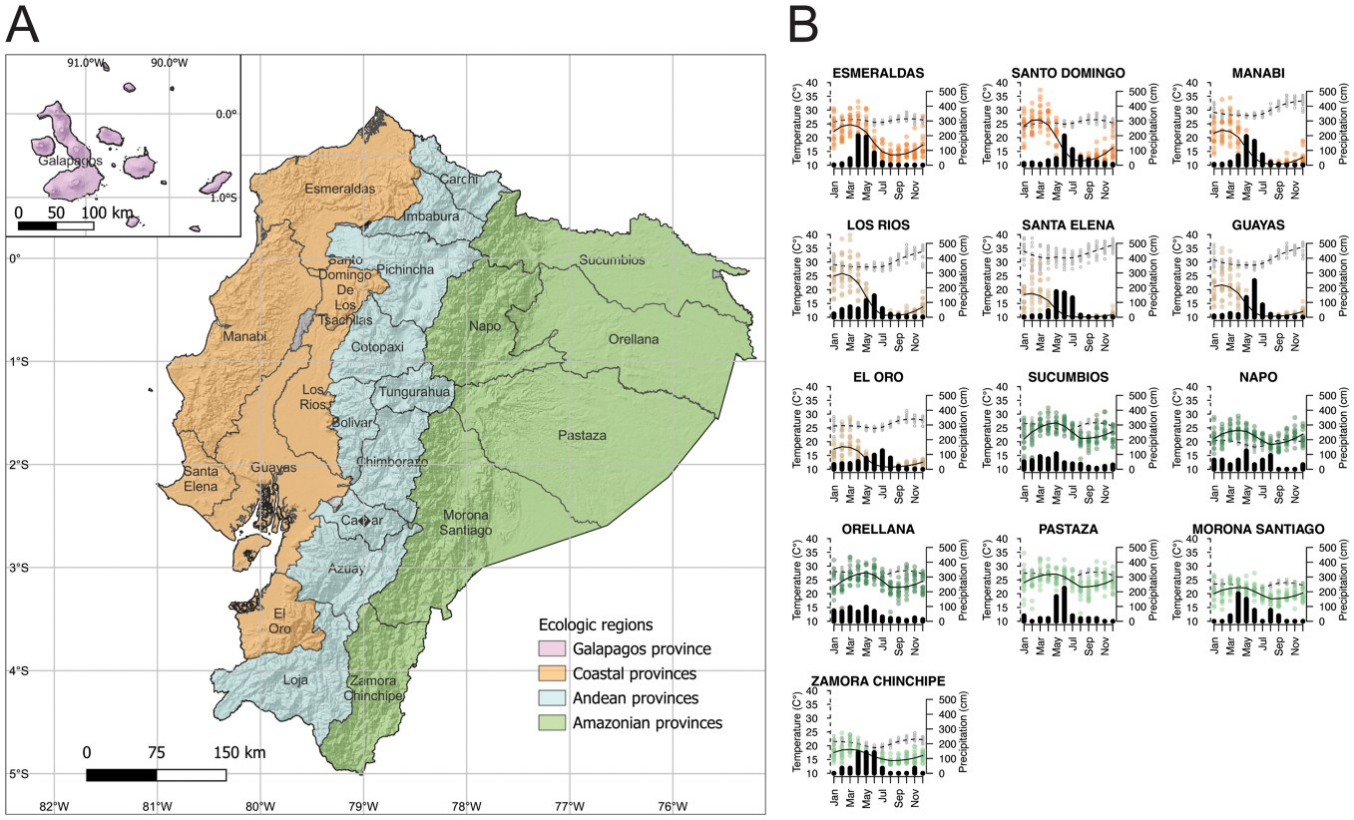
Geography and arbovirus seasonality of Ecuador. **(A)** Map of Ecuador, colored to indicate geographic and climate regions (coast, mountains, vs. Amazon). The base layer of the map is publicly provided by INEC [41]. **(B)** Temperature (grey dots and dotted lines), precipitation (dots colored by region, solid black line), and monthly distribution of arbovirus cases (black histograms). Annual province-level temperature and precipitation data from 2005–2019 were derived from the Universidad Tecnológica Equinoccial (UTE)/Centro de Investigación en Salud Pública y Epidemiología Clínica (CISPEC) database. Trend lines were fitted using local weighted regression (LOESS) models [42,43]. Monthly arbovirus case distributions for dengue, Zika, and chikungunya from 2014 to 2017 are derived from the ViEpi dataset.

Ecuador is a country in South America of 283,561 square kilometers and a total population size of just over 17 million people. Ecuador is divided down the center by the Andes mountains; provinces in this region are at an altitude too high to support *Aedes* populations and DENV transmission (Fig 1A). To the west of the Andes are the coastal lowlands, which border the Pacific Ocean, are densely populated, have high temperatures and humidity, and distinct wet and dry seasons. The provinces of Esmeraldas, Manabí, Santa Elena/Guayas, Los Ríos and El Oro consist primarily of lowland regions while Santo Domingo de los Tsáchilas/Pichincha contain both lower and higher elevation areas. Esmeraldas is the only province north of the equator, and both Esmeraldas and higher-elevation Tsáchilas/Pichincha have lower and more stable year-round temperatures than other provinces in this region (Fig 1B). In all coastal provinces, the rainy season extends from November through May, with the highest number of arboviral disease cases occurring between March and July. Six of the eight cities in Ecuador with populations larger than 200,000 are on the coast, including the major port cities Guayaquil (Guayas) and Manta (Manabí). The six eastern provinces of Ecuador are part of the Amazon, with a tropical climate but lower population density and no cities with more than 120,000 people. Precipitation is high year-round, with slightly more rainfall from March to July and in the northern compared to the southern Amazon provinces. Consistently, there is less seasonality of arbovirus transmission in the Amazon than the coastal provinces. The Galapagos Islands constitute the fourth region of Ecuador and while cases are sporadically reported, there is little evidence of sustained transmission.

### Age-stratified dengue incidence data and catalytic models

Dengue incidence was estimated from national surveillance data collected by INEC. The INEC dataset included all discharge data for hospitalized cases classified as vector-borne viral diseases and/or hemorrhagic fevers by International Statistical Classification of Diseases (ICD). Data were available from 2000 to 2019 and were grouped by age, sex, and province. For analysis, ICD codes A90 (Classic Dengue Fever) and A91 (Dengue Hemorrhagic Fever) were grouped as dengue cases (1997 WHO criteria for severe dengue disease). Data were binned into 18 age groups (<1, 1–4, 5–9, 10–14, 15–19, 20–24, 25–29, 30–34, 35–39, 40–44, 45–49, 50–54, 54–59, 60–64, 65–69, 70–74, 75–78, and ≥80 years of age) for analysis. To estimate dengue incidence, we used provincial demographic data as our denominator. National census data from 1991, 2000, and 2010 were available for each province and age group; we used interpolation to estimate the age-specific population size for each year from 2000 to 2019. There were sufficient case counts in all provinces in the coastal region (Esmeraldas, Manabí, Guayas/Santa Elena, El Oro, Pichincha/Santo Domingo de los Tsáchilas, Los Ríos) to enable province-level DENV FoI estimation. Although Santa Elena was separated from Guayas province and Santo Domingo de los Tsáchilas was separated from the Pichincha province in 2007, we leave them as combined provinces in our analyses to maintain consistency across years. Due to lower absolute case counts for the Amazon provinces (a median of < 4 cases per age group and year, leading to high noise in the data), we combined the case data for Sucumbíos, Napo, Orellana, Pastaza, Morona Santiago, and Zamora Chinchipe provinces. To evaluate the relative contribution of each age group to the total yearly incidence, we plotted the age-stratified dengue incidence normalized by the total incidence in each year to account for differences in absolute epidemic magnitude. Data were grouped into two periods (2000–2009 and 2010–2019) to visualize temporal changes in transmission. To compare distributions, data were fit with a LOESS curve with 95% confidence intervals. We used the age-adjusted dengue incidence data to estimate medians and interquartile range of ages by province for each 10-year period.

We used catalytic models to estimate historical and current FoI of DENV for each province based on age-stratified incidence using the method and code described by Rodriguez-Barraquer et al. [9], as follows. Given that cases were not serotyped but all four serotypes are known to circulate in Ecuador, we assumed equal FoI for the four DENV serotypes, as in previous dengue incidence models where case data by serotype were not available [9,12]. Thus, the fraction susceptible to all four DENV serotypes at age *a* and time *t* is:

$$x\left(a,t\right)=e^{\int_{0}^{a}-4\lambda \left(t-\tau \right)d\tau }$$

Where *λ*(*t*) is the average serotype-specific FoI and *τ* is the time since birth. We assumed the surveillance data reflect incidence of second DENV infections, as has been assumed previously using similar models [9,11,13,15]. This assumption is supported by decades of evidence that prior immunity to DENV is a strong risk factor for severe dengue disease in both Asia and the Americas [44–46], and that those experiencing second infections are much more likely to be hospital-seeking than primary or tertiary/quaternary infections and thus more likely to be detected by the surveillance system [18,47]. Models testing the relative contribution of primary, secondary, tertiary, and quaternary infections to case distributions further support this assumption [12,14]. We thus assume the at-risk population consists of individuals with primary DENV immunity, i.e., individuals infected with one DENV serotype but still at risk of infection with any of the other three serotypes. The fraction that has been infected with one serotype and remain susceptible to the other four serotypes is:

$$z_{1}\left(a,t\right)=4\;\left(e^{-\int_{0}^{a}3\lambda \left(t-\tau \right)d\tau }\right)\left({1-e}^{-\int_{0}^{a}\lambda \left(t-\tau \right)d\tau }\right)$$

Thus, the age-specific incidence for DENV infection is assumed to reflect the primary immune group experiencing their second infection with any of the other serotypes:

$$I\left(a,t\right)=3\lambda (t)z_{1}(a,t)$$

The expected number of reported cases of dengue at age *a* and time *t*, *Λ*(*a*, *t*), is a function of age-specific infection incidence *I(a,t)*, as well as the population size *P(a,t*) and reporting rate *ϕ*(*t*):

$$\Lambda \left(a,t\right)=I(a,t)P(a,t)\phi (t)$$

The reporting rate accounts for annual differences in the reporting rate of cases (not all cases are reported clinically) and the proportion of cases that are symptomatic among all DENV infections (not all infections cause cases). The variable *ϕ*(t) is assumed to be constant across ages within a given year. We assume the observed number of reported cases at age *a* and time *t*, *C(a,t)*, follows a Poisson distribution with the likelihood:

$$L\left(C|\lambda ,\phi \right)=\prod_{t}\prod_{a}\frac{{\Lambda \left(a,t\right)}^{C(a,t)}e^{-\Lambda (a,t)}}{C\left(a,t\right)!}$$

Models were fit with Rstan [48] using the Bayesian Markov Chain Monte Carlo framework in R [49]. We ran four independent Markov chains with 10,000 iterations of warmup followed by 20,000 iterations for estimating parameters. Parameters were evaluated for convergence using the split $\hat{R}$ statistic (values of 1 were assumed to indicate convergence) as well as visual inspection. All FoI estimates are shown as means with 95% credible intervals. The original authors of this code [9] note that given the large numbers for incidence, credible intervals may be systematically underestimated.

For our didactic example of CHIKV and ZIKV FoI, we assumed the age-specific incidence of chikungunya and Zika cases reflect the fully susceptible group experiencing a primary infection:

$$I\left(a,t\right)=\lambda (t)x(a,t)$$

All other equations are the same for CHIKV and ZIKV.

### Comparison of dengue, chikungunya, and Zika incidence by province

The ViEpi dataset included de-identified individual case information for 2014 (November to December), 2015 (full year), 2016 (full year), and 2017 (January to April) meeting the following case definitions: (1) Dengue with Warning Signs or Severe Dengue, (2) chikungunya, and (3) Zika. The dataset included the following information for each case: age, sex, nationality, home district, province, canton, parish, date of illness, whether and how the case was confirmed (laboratory confirmation, clinical, contact case), hospitalization status, and outcome of case. Cases were grouped by the same age categories as indicated above for the INEC dataset. We use suspected, and not just laboratory-confirmed cases, as there is an age-specific bias in the cases that were confirmed. Notably, the incidence of chikungunya and Zika cases in infants and for Zika, reproductive age individuals, is higher than would be expected, possibly due to greater confirmation in these age groups. We used the ViEpi dataset (n = 41,777 total cases) to estimate: 1) province-level differences in age-stratified disease incidence of dengue, chikungunya, and Zika (2015–2016 data), and 2) province-level total monthly arbovirus case distributions (2014–2017).

### Entomological data

*Aedes aegypti* presence was measured as part of the INSPI-SENESCYT Project “National Vigilance and Early Warning System for Vector Control of Dengue-Yellow Fever” (SATVEC PIC-12-INH-002). Systematic data collection procedures were used to ensure a comprehensive representation of mosquito populations across different geographic regions, allowing for a more robust and accurate analysis of their distribution and characteristics. *Aedes aegypti* mosquito samples were collected from 2013 to 2018 at 1785 collection sites in Ecuador and encompassing 23 provinces and 43 cantons (the second-level subdivision below provinces). Sampling sites ranged in elevation from 0 to 1650 meters above sea level. In total 946 records correspond to collections during the rainy season and 839 during the dry season. Geographic coordinates were obtained for all collections. Both immature and adult mosquito samples were collected from field locations and transported to the laboratory for rearing and species identification.

### Species distribution mapping using maximum entropy models

We used the confirmed vector presence data in maximum entropy models to evaluate the species distributions for *Aedes aegypti* across Ecuador using the MaxEnt [50]. The maximum entropy model uses environmental and social layers to estimate the probability of presence of a species within each raster unit to predict the distribution of a species across the map [51,52]. MaxEnt was selected because it requires only presence data for the species being studied, has been widely used and produces consistent results even with a limited number of presence points, and has a GIS interface within the TerrSet GIS software (Clark Labs). This method has been used previously for characterizing the distribution of vectors like *Aedes albopictus*, *Aedes aegypti*, and arboviral diseases including dengue [53–56]. Variables previously identified as predictors of arbovirus transmission were included as layers in the model: two physiographic variables (elevation, slope), eight demographic and social variables derived from available census data (literacy, employment, overcrowding, population density, housing condition, water access, sewerage, garbage collection), and three environmental/climatic variables (precipitation, Daytime Land Surface Temperature, and the Enhanced Vegetation Index). All variables were downscaled at a resolution of 1 hectare (100m x 100m), corresponding to the most detailed layers in the INEC census database. In contrast with other models in Ecuador that have used the WorldClim database [30,55], we derived our environmental geodatabase directly from EarthData [57]. To process the spatiotemporal series of monthly images from 2010 to 2018 as environmental variables for analysis in the MaxEnt model, we applied an orthogonal transformation of n-dimensional image data using principal component analysis (PCA). This produced a new set of images (components) that are uncorrelated with one another and ordered with respect to the amount of variation (information) they represent from the original image set. These techniques were implemented in TerrSet. As we were working with a cube X-Y-T, the PCA could be adapted to the various dimensions. For the three climatic parameters (temperature, precipitation, and vegetation) we used two PCA components in T-mode and 2 PCA loadings in S-mode. Using this approach, the model was not affected by layers that would not be explicative.

We trained the MaxEnt model on 75% of presence points, with 25% for validation, in 10 bootstrap replicates each of 500 iterations. Model goodness-of-fit and prediction was evaluated using receiver operating characteristic (ROC) analyses and binomial tests of omission. The area under the ROC curve (AUC) was 0.92 for the training set and 0.916 for the test set, with a standard deviation of 0.003. For our binomial test of omission, we applied a logistic threshold of 0.416 that equals training sensitivity and specificity, yielding a fractional predicted area (used instead of the commission rate because there is no absence data) of 0.132, a training omission rate of 0.132, and a test omission rate is 0.156.

## Results

### Age-distributions of dengue incidence vary by province and over time

**Fig 2:**
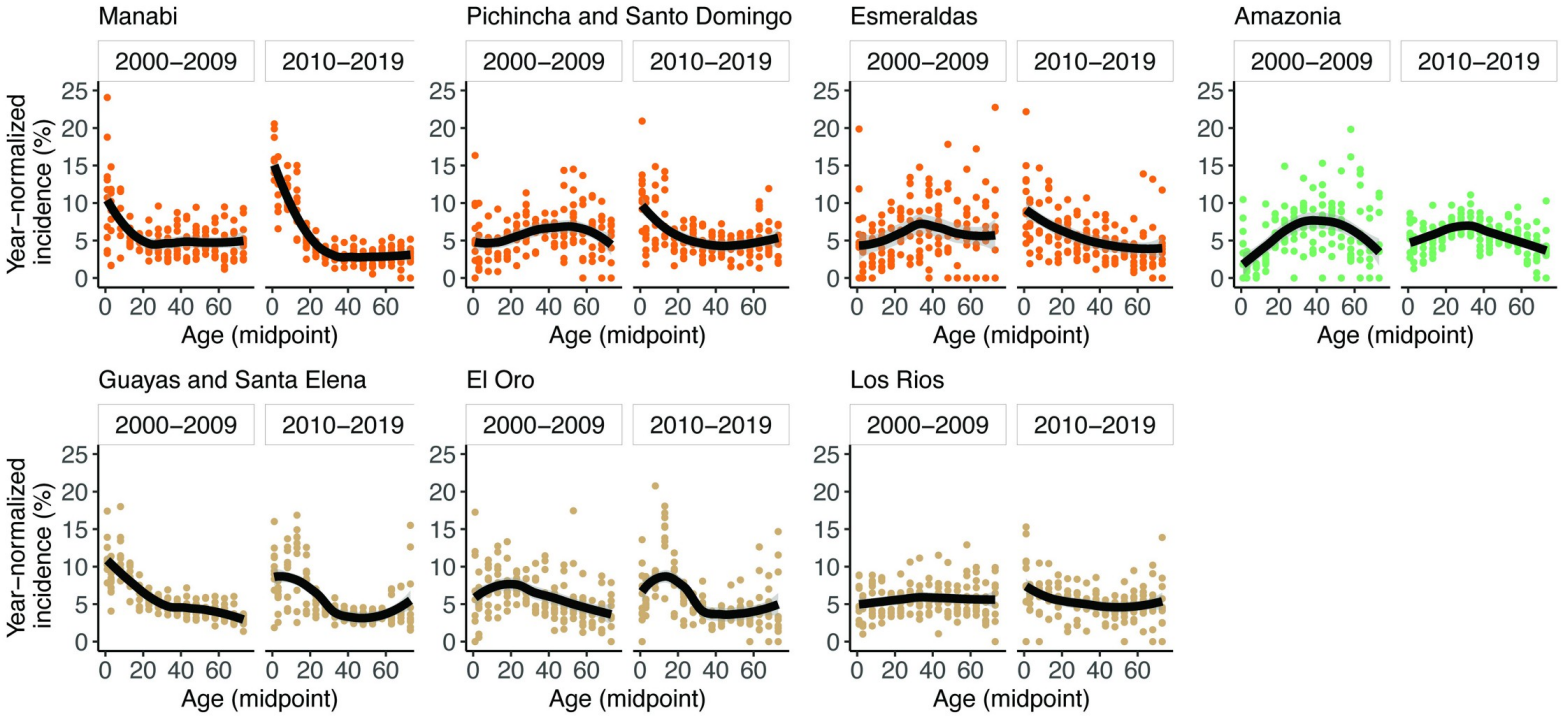
Age-specific incidence of dengue, normalized by year, for each province and period. Data are fitted using LOESS regression with 95% confidence intervals. Data are from the INEC database.

We measured temporal and geographic differences in DENV transmission intensity by estimating age-stratified dengue incidence for each province between 2000–2019. We estimated dengue incidence using national surveillance of Dengue Fever and Dengue Hemorrhagic Fever cases and interpolated age-specific province-level population size data. For data visualization, data were grouped into two 10-year periods, 2000–2009 and 2010–2019 (Fig 2). The median age of the population in each province ranged from 20–26 throughout the 20-year period, with the lowest median age in the Amazon and highest age in Guayas/Santa Elena and Pichincha/Santo Domingo (Table 1). The first decade of dengue incidence data had different features compared to the second decade of data. For example, from 2000 to 2009, the population-dense coastal provinces of Guayas/Santa Elena, El Oro, and Manabí had the lowest median age of dengue cases (28–36 years) whereas in provinces with higher elevation or that were more rural and inaccessible (Esmeraldas and the Amazon), the median dengue incidence occurred at older ages (41–43 years). All provinces except Guayas/Santa Elena experienced a drop in median dengue incidence between 2000–2009 and 2010–2019, with the largest change in Manabí (by 17 years), as well as large changes in Esmeraldas, the Amazon, and Pichincha/Santo Domingo (6–9 years).

**Table 1:** Median and interquartile range (IQR) of population-adjusted dengue incidence by province, from 2000–2009 and 2010–2019. Median and IQR of the population during the same period are shown for reference.

Province	Incidence: median (IQR)		Population: median (IQR)	
	2000–2009	2010–2019	2000–2009	2010–2019
Manabí	36 (13–58)	19 (8–48)	24 (12–41)	25 (12–43)
Pichincha/Santo Domingo	43 (25–60)	35 (14–59)	26 (13–42)	26 (13–42)
Esmeraldas	41 (25–59)	32 (14–56)	22 (11–39)	22 (11–40)
Amazon	43 (27–58)	37 (22–57)	20 (9–36)	21 (10–37)
Guayas/Santa Elena	28 (11–51)	28 (13–58)	26 (13–42)	26 (12–43)
El Oro	32 (16–51)	28 (13–57)	25 (12–42)	26 (13–44)
Los Ríos	41 (22–60)	39 (18–62)	24 (11–41)	25 (12–42)

### Models of age-stratified dengue incidence reveal historical and geographic changes in DENV transmission

**Fig 3:**
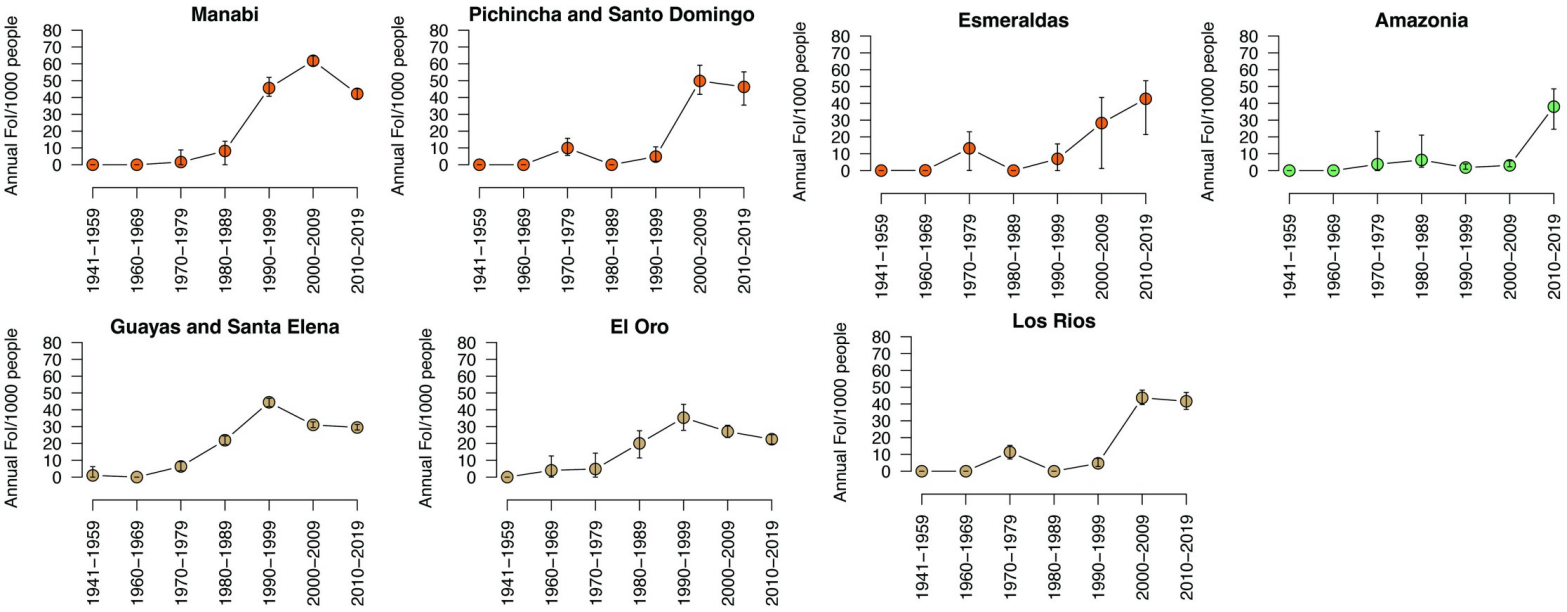
DENV FoI estimates for provinces across Ecuador. Point estimates are colored by geographic region, 95% credible intervals are shown.

We assumed that the age distributions of dengue incidence by province were due to both current and historical transmission of DENV in each region. We estimated the FoI of DENV for 10-year increments back to 1941, covering the earliest period when individuals in the dataset were alive. We modeled annual age-stratified dengue incidence directly using Bayesian models based on the method described by Rodriguez-Barraquer et al. [9]. For many pathogens, the age-distribution of disease reflects the average age at which individuals experience their first infection [58]. However, secondary DENV infections are more likely to cause symptoms and disease severe enough to elicit a hospital visit than either primary or tertiary/quaternary infections [12,14,18,47]. Thus, most cases captured by surveillance likely represent secondary DENV infections, both in Asian and the Americas [13]. Consistent with prior dengue incidence analyses, we thus assume that the age-specific incidence of dengue is driven by those experiencing their second DENV infection [9,11–15,44–47]. We observed that all provinces and regions had minimal DENV transmission for the period of 1941 to 1979 (Fig 3). Consistent with this observation, from 1946 to 1970, continent-wide *Aedes* eradication campaigns were underway. A program was implemented in 1958 to specifically monitor *Aedes aegypti* [3]. No dengue cases were recorded in Ecuador during this time, but *Aedes* successfully reinfested the cities in Guayas and Manabí between 1977 and 1985. Our FoI models estimated that between 1980 to 1989, DENV transmission intensity began to rise in Guayas/Santa Elena and El Oro (Fig 3). Then from 1990–1999, high DENV transmission was observed in Guayas/Santa Elena, El Oro, and Manabí. These results are consistent with the first documentation of dengue in Ecuador in 1985 and major outbreaks in Guayas in 1988, 1990, and 1993. At this time, dengue was re-emerging across the Americas, and the introduction of multiple serotypes resulted in the first observed cases of Dengue Hemorrhagic Fever/Dengue Shock Syndrome [3,4,59]. The largest and earliest dengue outbreaks were concentrated in the large coastal cities in Guayas and Manabí, consistent with our estimated timeline for the re-introduction of DENV to Ecuador.

Other regions with suitable ecological conditions for DENV transmission in Ecuador had not recorded major epidemics. Consistent with these epidemiological data, we found that the early rise of DENV in the southern coastal provinces was not observed in the northern coastal province of Esmeraldas, the higher elevation coastal provinces of Los Ríos and Pichincha/Santo Domingo de los Tsáchilas, or the Amazon provinces, which had very low levels of DENV transmission from 1990–1999 (FoI: ≤10 per 1000 people). These provinces had fewer dense urban settings and were more isolated before the year 2000, with limited road infrastructure. Pichincha/Santo Domingo, Los Ríos, and Esmeraldas had high DENV transmission intensity in 2000–2009 and 2010–2019 (FoI: 40–50 per 1000 people). The Amazon provinces were most delayed, experiencing a rise in DENV transmission only in the last 10 years (40 per 1000 people). Thus, we observe the provinces of Ecuador at different stages of endemicity, with some only recently experiencing increases in transmission intensity.

### Age-distributions of recently emerged arboviral diseases reflect risk across ages

**Fig 4:**
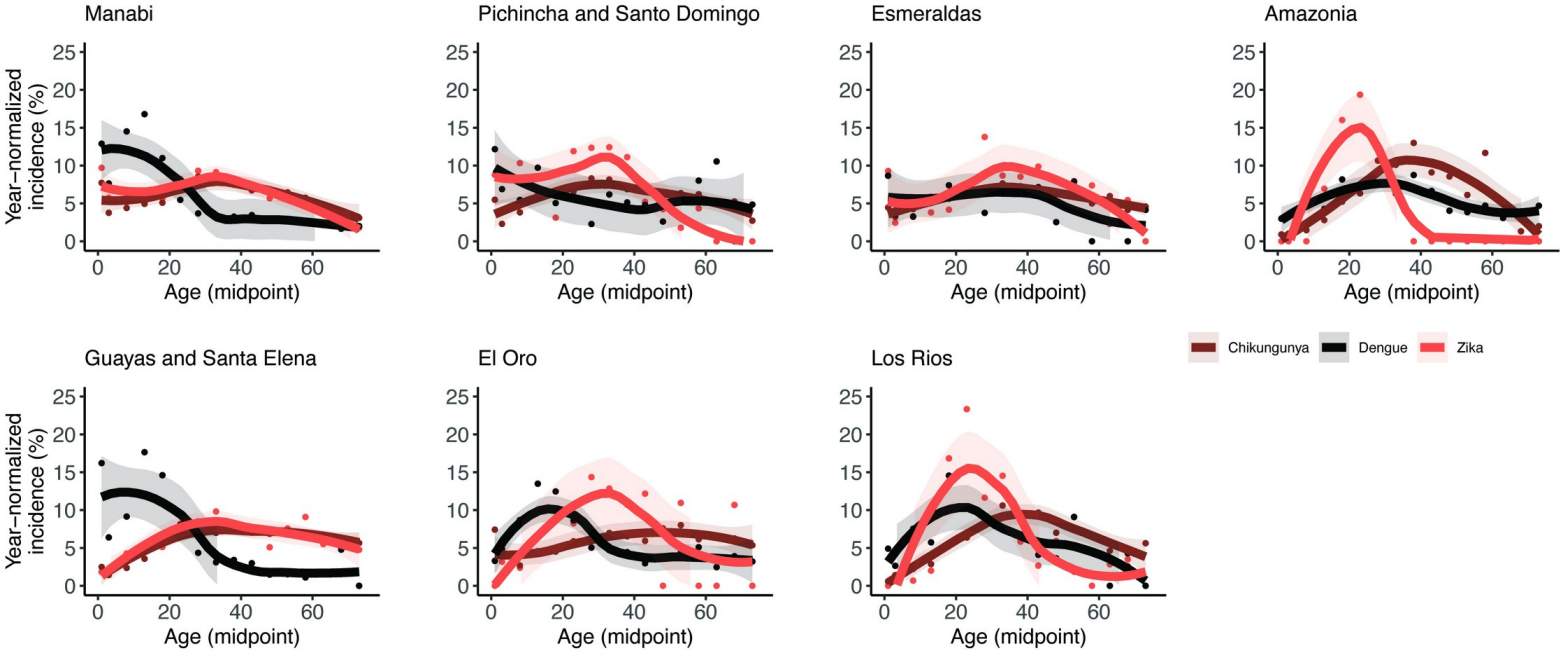
Age-specific incidence of laboratory-confirmed and suspected Dengue with Warning Signs/Severe Dengue, chikungunya and Zika for each province in 2015–2016. Data are fitted using LOESS regression with 95% confidence intervals. In some provinces, unusual distributions for Zika suggest case-confirmation focused on specific age groups.

Chikungunya caused a major outbreak in Ecuador in 2015, soon followed by a national Zika epidemic in 2016 [8,35]. We expected that the age distributions of these new arboviral diseases would be similar across provinces, consistent with their recent introduction. We measured age and disease-specific incidence using data from a separate surveillance system, ViEpi, consisting of laboratory-confirmed and suspected cases of chikungunya, Zika, and Dengue with Warning Signs/Severe Dengue observed between 2015 and 2016. Across provinces, Zika and chikungunya incidence was mid-aged centered, consistent with recent emergence, although in some regions, Zika incidence was more concentrated in reproductive-age individuals, likely due to a bias in laboratory confirmation and reporting of cases (Fig 4). Notably, the age-distributions of chikungunya and Zika incidence more closely resembled the age distributions of dengue incidence in Esmeraldas, Los Ríos, Pichincha/Santo Domingo, and the Amazon, but were starkly different in Guayas/Santa Elena, El Oro, and Manabí. These findings further support the hypothesis that heterogeneity in the age distribution of dengue incidence across Ecuador reflects historical differences in the time of DENV emergence and the transition to endemicity.

**Fig 5:**
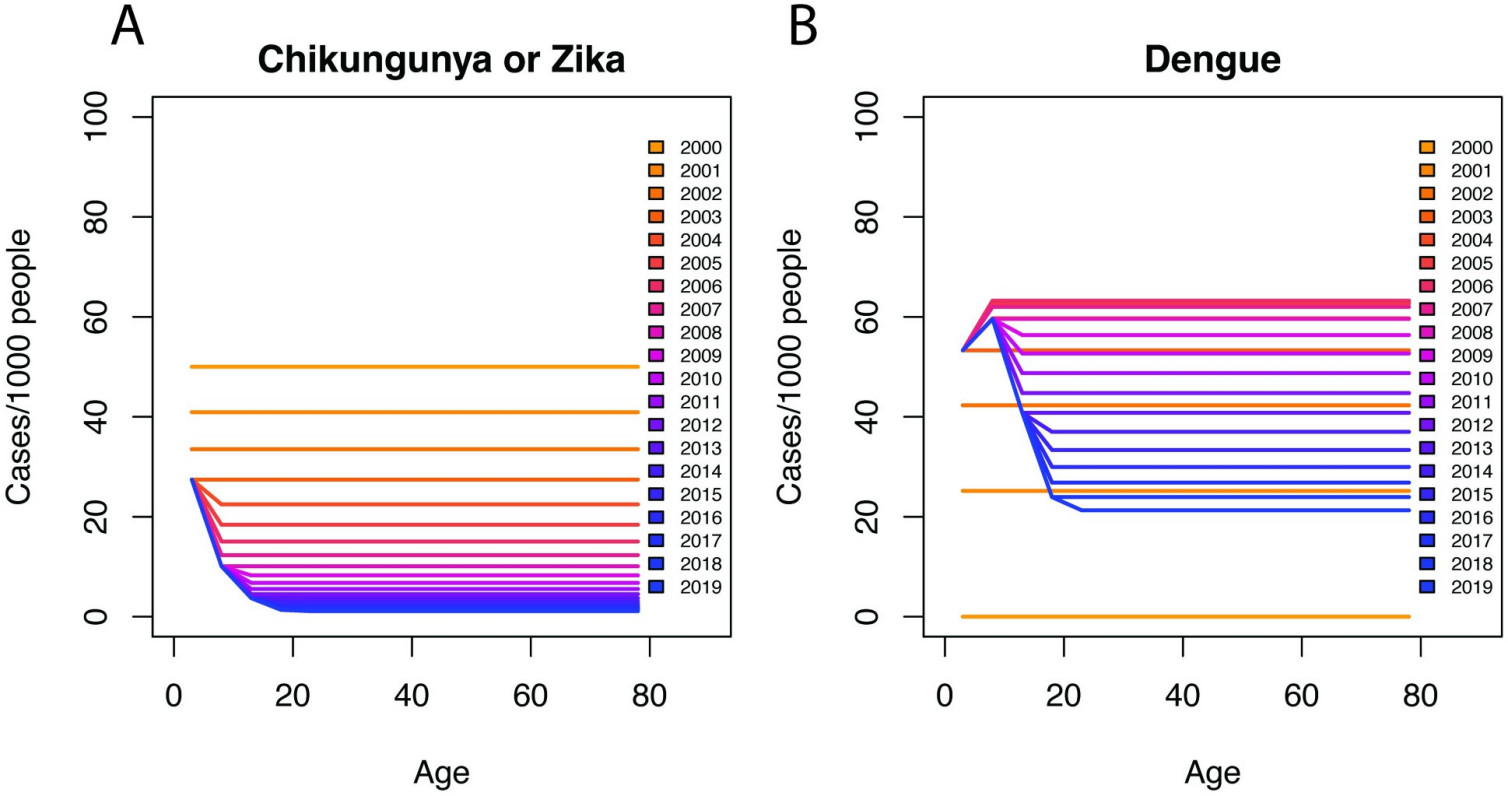
Age-stratified incidence of different arboviral diseases by time since emergence. Expected changes in age-specific incidence for a viral disease that emerged 20 years ago with an annual FoI of 50/1000, assuming the first infection causes disease (like chikungunya or Zika) vs. when there are four circulating viruses that emerged 20 years ago each with an annual FoI of 50/1000 and the second infection causes disease (like dengue).

The age-specific incidence distribution for arboviruses such as chikungunya and Zika, where a first infection causes disease, is expected to differ from dengue, where a second infection is most likely to cause disease, as illustrated using a theoretical modeling example (Fig 5). For all three diseases, as years pass since emergence, the age distribution of incidence shifts from mid-age distributed to younger ages as older individuals become immune and disease only affects young, naïve individuals. While for chikungunya and Zika, explosive epidemics are observed immediately across age groups, for dengue, it takes multiple years before high incidence is observed across age groups as individuals experience second infections. Further, the shape of the age distribution of cases at endemicity is distinct for dengue compared with chikungunya and Zika, reflecting these differences in the susceptible population.

### Determinants of *Aedes* risk help explain geographic variation in DENV transmission intensity

**Fig 6:**
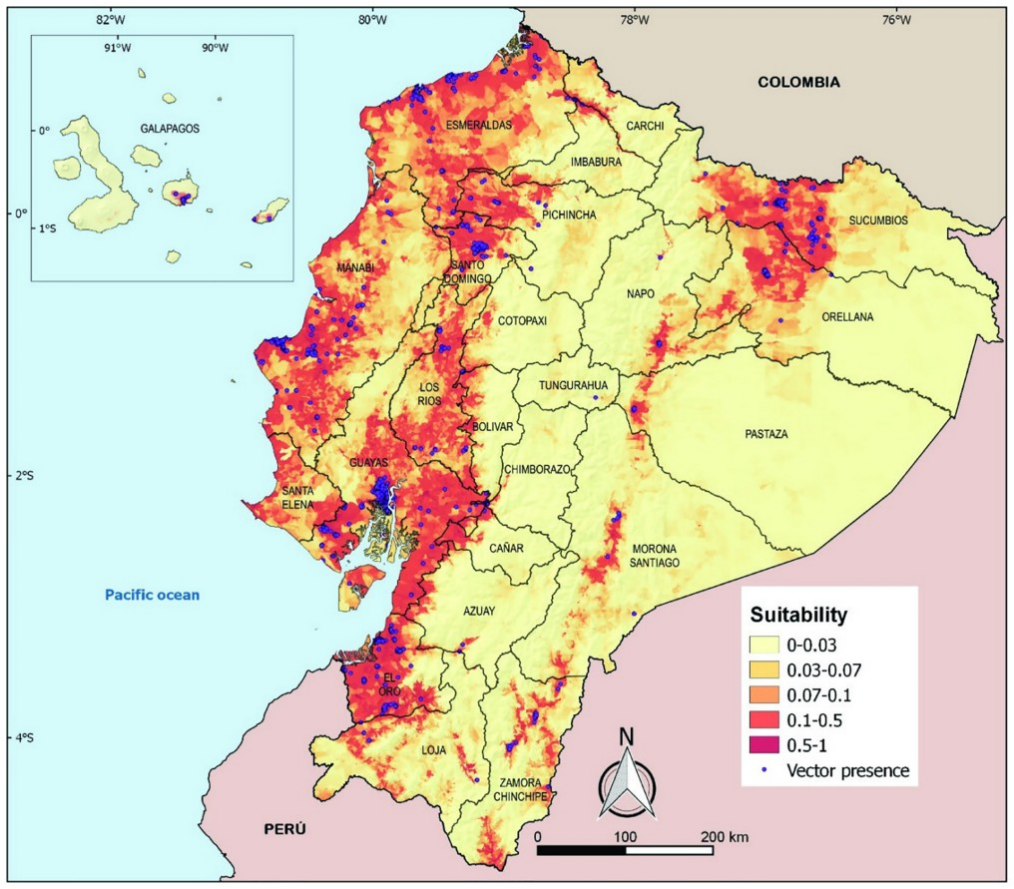
Maximum entropy geographic models using environmental and demographic variables to predict *Aedes aegypti* presence data. The base layer of the map is publicly provided by INEC [41].

To evaluate which environmental and demographic factors were associated with the observed geographic variation in DENV transmission intensity, we used maximum entropy models to identify predictors of *Aedes aegypti* presence data. We sampled immature and adult stages of *Aedes aegypti* mosquitoes in 1785 distinct collection sites for a total of n = 11,693 distinct samples (Fig 6). The percentage of sites with presence of *Aedes aegypti* in the coastal provinces was higher (35.9%) than in the Amazon provinces (13.4%) but *Aedes aegypti* was present at higher altitudes in the eastern Amazon basin (1650 meters) than the coastal region (1000 meters). In our maximum entropy models, 55.5% (123,087 km^2^) of the territory was suitable (medium or high risk) for *Aedes aegypti*. Most of the coastal lowlands had medium risk for *Aedes aegypti*, with high-risk zones in more populated areas. Almost all of Guayas and El Oro were at high risk, while large sections of Manabí, Esmeraldas, Pichincha/Santo Domingo, and Los Ríos were also at high risk. Much of the Amazon was predicted to be at low risk, although the northern Amazon and Amazon regions closer to the Andes were also at medium to high risk for *Aedes aegypti*. The variables that most strongly predicted *Aedes aegypti* presence included population density (63.3%), garbage collection (14.3%), and elevation (11.2%) (Table 2). *Aedes aegypti* risk was associated to a lesser extent with water access, vegetation, sewage connection, precipitation, house quality, and other variables.

**Table 2:** Variable importance of risk factors predicting *Aedes aegypti* presence data.

*Aedes* model	
Variable	Percent contribution
Population	63.3
Garbage collection	14.3
Elevation	11.2
Water access	3.6
Vegetation	1.8
Sewage connection	1.6
Precipitation	1.0
Housing quality	0.7
Other variables	2.5

## Discussion

In this study, we described changes in the transmission intensity of DENV across ecologically diverse provinces of Ecuador over nearly 80 years and identified multiple environmental, demographic, and social factors associated with the distribution of the vector. Our estimates of the FoI of DENV and risk for the DENV vector complement and enrich the documented record of DENV emergence across Ecuador and inform changes driving the expansion of arboviruses globally.

We found that DENV transmission in Ecuador increased as dengue resurged across the Americas, first in the southern coastal provinces with major port cities, then to other connected regions in the coastal lowlands. Consistent with our findings, a study in Brazil using catalytic FoI models documented a shift in dengue incidence from adults to children in the decades following emergence, a pattern expected of dengue as it becomes endemic [13]. Our analyses suggest that DENV only spread to more isolated regions of Ecuador in the 21^st^ century, possibly driven by increased human movement, as has been documented across the Americas [60,61]. Starting near the turn of the 21^st^ century, the government of Ecuador began to heavily invest in roads and infrastructure. The new roads increased connectivity to previously isolated regions, including major cities in Esmeraldas and the Amazon, as well as to more rural areas in all provinces [62,63]. These changes may have driven the uptick in DENV FoI in Esmeraldas and the Amazon, which were not previously considered high risk areas. In Esmeraldas, connectivity first increased with Colombia, consistent with the observation that the DENV serotypes circulating in Esmeraldas between 2010–2014 more closely matched those circulating in Colombia than the rest of Ecuador [34,36,62]. A more recent study demonstrated dissemination of DENV1 and DENV2 from urban centers to more isolated communities in the Esmeraldas province [36]. Seroprevalence data from Esmeraldas demonstrates longer-term circulation of DENV and more recent introduction of CHIKV, with rural areas more likely to be only positive to DENV while those in urban areas were more likely to be exposed to both viruses, suggesting emerging pathogens take longer to reach remote areas [64]. Consistent with this observation, CHIKV caused an outbreak in rural and highly isolated communities in Esmeraldas only in 2019, long after the major chikungunya epidemic across Ecuador in 2015 [65]. As previously low-population rural areas become increasingly urbanized and connected to other rural areas and to major cities, their role in maintaining transmission and helping reseed epidemics is expected to increase [36,66]. Further, lack of infrastructure often tracks with rapid urbanization, and may result in limited water access, garbage collection, and sewage, which are all local arboviral disease risk factors in Ecuador [23–27].

In total, we found that 55.5% of Ecuador currently has favorable conditions for *Aedes aegypti*, including the provinces we identify as experiencing increases in DENV transmission intensity in the last 10 years. Notably, we also found that higher elevation regions like Pichincha/Santo Domingo and Los Ríos experienced an increase in dengue, possibly due to changes in global temperature which are predicted to expand the range of *Aedes aegypti* into higher elevation areas of Ecuador [30,55,67]. Our geographic models show that the coastal provinces and Amazon have high risk areas for the vector, and that population density, garbage collection, and elevation explain the most variation in vector distributions. Our estimates of high vector risk in coastal Ecuador agrees with global dengue maps and are higher than global *Aedes* maps; however, our estimates suggest lower risk in parts of the Andes and Amazon than predicted by some other global models [10,54,68,69] and models of Ecuador, possibly due to use of a different immature and adult mosquito dataset [30,67]. The predictors we identified as important for explaining risk partially agree with previous mapping efforts, including poor housing infrastructure and population density [54]. We also found that elevation had a strong effect. Numerous studies in Ecuador [30,55,67] and globally [10,68,69] have shown that population density, precipitation, and minimum temperature are important determinants of dengue presence and transmission intensity. In one study, the effect of elevation was small once other variables were considered [10]; given strong correlation between these variables, especially in Ecuador, it may be that they are measuring similar phenomena.

The ecological conditions in regions where DENV has recently emerged may further increase DENV intensity. For instance, while in Guayas and Manabí, entomological surveys reveal low vector density during the dry season and larvae proliferation during the wet season [33], a household survey in Esmeraldas found more complex relationships between mosquito density, rainfall, and water storage practices [70]. In the Amazon, there is not as strong a distinction between the wet and dry seasons, potentially enabling more sustained arbovirus transmission year-round. The combined effects of Amazon ecological conditions, urbanization, and population growth are evident in the Amazon cities of Iquitos, Perú and Manaus, Brazil, where once *Aedes aegypti* and DENV were introduced and the cities were large enough, the conditions were primed for severe epidemics and sustained transmission [71]. In Iquitos, Perú which is mainly accessible by river barge, invasion of the *Aedes aegypti* species was facilitated by these river boats traveling from urban to peri-urban to rural areas [72]. For this reason, similar attention should be paid to changes in the Amazon provinces of Ecuador, which are increasingly affected by population growth, urbanization, and migration due to the expansion of the oil industry [73].

Our study has several limitations. We were unable to test whether changes in *Aedes* risk were temporally associated with changes in DENV transmission intensity, as data on ecological and mosquito density data were not available before 2013. Further, we were unable to determine changes in the serotype-specific intensity of DENV transmission due to lack of serotype-level case data. For our FoI models, we used cases classified as Dengue Fever or Dengue Hemorrhagic Fever by the ICD code, and not those classified as other viral or hemorrhagic fevers. Given that not all cases were laboratory confirmed, it is possible that some cases caused by other diseases were accidentally counted as dengue and included in our analyses. Additionally, our analyses depended on cases detected by surveillance, which are mostly hospitalized cases. In Ecuador, as in other places, hospitalized cases underestimate disease compared to passive surveillance and depend on local healthcare provider practices [74,75]. We were also unable to quantitatively measure the effect of changes in connectivity between regions and DENV transmission intensity due to lack of available data. Due to low numbers of cases, we were unable to measure transmission changes for each separate province in the Amazon. Finally, we were unable to analyze the impact of the COVID-19 epidemic on the FoI of DENV. Data collected on dengue are likely to be highly underreported in 2020 and 2021 and may have biases in age of reporting that would alter our interpretation of the data. Dengue epidemics returned throughout Americas in 2022, as the COVID-19 pandemic waned [76]. Why this happened, and whether the COVID-19 epidemic impacted dengue has been a topic of multiple other studies and could be the topic for of a future investigation in Ecuador if appropriate data sources could be identified.

Here, we tracked the changes and drivers of DENV transmission across Ecuador using over 20 years of longitudinal case data, detailed entomological measurements, catalytic models of DENV FoI, and maximum entropy models for species distribution modeling. We show that coastal provinces with large, connected cities experiencing the highest and earliest increase in DENV transmission intensity. Areas that remained isolated like the northern coast and the Amazon regions more recently experienced a rise in DENV transmission and endemicity. However, all provinces with suitable ecological conditions were affected by the chikungunya and Zika pandemics and had regions with high *Aedes* risk. Future local and national control strategies should consider the emergence and re-emergence of multiple arboviruses transmitted by *Aedes aegypti* as well as differences in the natural and built environments, including the rapid expansion of vector-borne diseases into areas that previously were minimally affected. As human movement, population growth, urbanization, and climate change intensify, new areas within diverse countries like Ecuador will be affected [30,55,67,68]. Vector surveillance combined with statistical prediction methods of climate and other factors can identify areas at high risk for arbovirus transmission and may serve early warning system for human dengue outbreaks [77,78]. Surveillance and vector control efforts may be further refined by learning from the historical dynamics for dengue, Zika, and chikungunya, recognizing the potential for novel emerging diseases to follow similar patterns and strategically developing control measures that consider interactions and shared risk factors for these diseases.
