# Correction: Increasing transmission of dengue virus across ecologically diverse regions of Ecuador and associated risk factors

**DOI:** 10.1371/journal.pntd.0013993

**Published:** 2026-02-12

**Authors:** Leah C. Katzelnick, Emmanuelle Quentin, Savannah Colston, Thien-An Ha, Paulina Andrade, Joseph N. S. Eisenberg, Patricio Ponce, Josefina Coloma, Varsovia Cevallos

An additional affiliation is missing for the fifth author. Paulina Andrade is also affiliated with the Biotechnology Department, School of Environmental Sciences, Universidad San Francisco de Quito, Quito, Ecuador.
